# Analysis of 55 patients with multiple endocrine neoplasia type 1-associated insulinoma from a single center in China

**DOI:** 10.1186/s13023-022-02370-1

**Published:** 2022-06-13

**Authors:** Yuan Zhao, Jie Yu, Yiwen Liu, Lu Lyu, Fan Ping, Lingling Xu, Wei Li, Ou Wang, Qiang Xu, Wenming Wu, Huabing Zhang, Yuxiu Li

**Affiliations:** 1 Department of Endocrinology, Key Laboratory of Endocrinology, Ministry of Health, Peking Union Medical College Hospital, Peking Union Medical College, Chinese Academy of Medical Sciences, Beijing, 100730 China; 2 Department of General Surgery, Peking Union Medical College Hospital, Peking Union Medical College, Chinese Academy of Medical Sciences, Beijing, 100730 China; 3 Chinese Academy of Medical Sciences, 1 Shuai-Fu-Yuan Wangfujing, Dongcheng District, Beijing, 100730 China

**Keywords:** Multiple endocrine neoplasia type 1, Insulinoma, Hypoglycemia, Hypercalcemia

## Abstract

**Objective:**

To investigate the clinical characteristics of patients with multiple endocrine neoplasia type 1 (MEN1)-related insulinoma and their relationship with specific biochemical changes and to summarize the features of treatment options for the Chinese population with this disease and the impact on long-term prognosis.

**Methods:**

“MEN1” and “insulinoma” were used when searching the Peking Union Medical College Hospital (PUMCH) medical record retrieval system to obtain clinical information about patients. We identified patients diagnosed with MEN1-associated insulinoma based on endocrinological, radiological, and pathological examinations, and subsequently analyzed their clinical data.

**Results:**

A total of 55 patients with MEN1-associated insulinoma were included, including 29 (52.7%) men and 26 (47.3%) women. The parathyroid gland was the most commonly affected (78.2%), followed by the pituitary gland (69.1%) and adrenal gland (16.4%). Insulinoma was the first manifestation of MEN1 in at least 23.6% (13/55) of patients. Nineteen (34.5%) patients presented with initial symptoms of hypoglycemia before the age of 22 years. Among the 24 Patients with high serum calcium (Ca) had significantly lower serum insulin levels than those with normal serum Ca levels (*p* < 0.001) during hypoglycemic episodes. However, serum C-peptide level at 0.5 h and serum insulin level at 1 h was higher in patients with hypercalcemia than in patients with normal serum Ca levels in the oral glucose tolerance test (OGTT), although the differences were not statistically significant. Multifocal pancreatic neuroendocrine tumors (pNETs) were present in 38 (69.1%) patients; most of them (55.6%, 20/36) underwent multiple enucleations, and 45% (9/20) had a postoperative recurrence. Five patients (10%) who underwent distal pancreatectomy developed pancreatic insufficiency after an average of seven years. patients who underwent genetic testing, 23 (95.8%) were positive for MEN1 mutation, with mutations most commonly found in exons 2 (21.7%) and 3 (13%).

**Conclusions:**

In our study, the rates of postoperative recurrence and long-term complications in patients with MEN1 with multifocal pNETs were significantly different from those in other international centers and might be related to the choice of surgical method. In addition, elevated serum Ca levels in patients with primary hyperparathyroidism may affect insulin secretion.

**Supplementary Information:**

The online version contains supplementary material available at 10.1186/s13023-022-02370-1.

## Introduction

Multiple endocrine neoplasia type 1 (MEN1) is a rare (prevalence 1–10/100,000) inherited multi-tumor syndrome that affects specific neuroendocrine organs and non-endocrine tissues, principally the parathyroid glands, gastro-entero-pancreatic (GEP) tracts, and anterior pituitary [1–3]. Insulinomas are the second most common neuroendocrine tumors of the functional GEP tract (GEP-NETs) in MEN1 after gastrinomas, affecting approximately 10–15% of patients with MEN1, and they often develop in patients aged 10–39 years, earlier than sporadic insulinomas [4]. Currently, surgical resection is the only curative treatment, and the choice of surgical method is associated with a short-term cure for hypoglycemia and the risk of long-term recurrence.

Parathyroid adenoma is the first clinical presentation in approximately 90% of cases, with a mean age of onset of 20–25 years and approximately 100% penetrance by the age of 50 years [4], manifesting principally as primary hyperparathyroidism (PHPT) but may present with elevated serum calcium (Ca) levels [3]. During nutritional stimulation, ionized calcium (iCa) has a critical role in β-cells by linking the metabolism of glucose and other secretagogues to the generation of signals that promote insulin secretion [5]. However, to our knowledge, no case series or epidemiological studies have described the characteristics of insulinomas in patients with MEN1 in the Chinese population and the effect of elevated serum Ca caused by PHPT on patients with insulinoma has not been studied.

This study aimed to analyze the clinical data of patients with MEN1-associated insulinoma treated at the Peking Union Medical College Hospital (PUMCH) from January 2002 to August 2021 and to explore the impact of biochemical changes caused by the presence of hypercalcemia on their clinical features to provide insights into screening, management, and follow-up strategies for this rare disease.

## Patients and methods

### Population

The data were retrieved from the PUMCH Medical Record Retrieval System. A total of 310 patients with a discharge diagnosis of MEN1 between January 2002 and August 2021 were identified using ICD-9/10 codes; 255 without insulinoma were excluded. Ultimately, 55 eligible patients were included in the analysis.

The diagnosis of MEN1 was established based on one of the following criteria [4, 6]: (1) presence of neoplastic diseases in at least two or more commonly affected organs affected in MEN1: parathyroid adenoma, endocrine pancreas, and pituitary adenoma (PA), (2) presence of neoplastic diseases in one of the three commonly affected organs affected in MEN1 and a first-degree relative affected by MEN1, (3) the identification of a germline mutation of the *MEN1* gene.

The presence of an insulinoma was based on randomly observed signs of hypoglycemia and the Whipple triad with concomitant biochemical endogenous hyperinsulinemic hypoglycemia (HH) according to clinical practice guidelines [4]. Localization methods included abdominal ultrasound, abdominal enhanced computed tomography (CT), pancreatic volume perfusion CT, octreotide imaging, glucagon-like peptide-1 receptor (GLP-1R) receptor imaging, and selective celiac arteriography. Pathological grading of insulinomas was based on the proliferation rate of tumor cells as determined by using the mitotic count and/or Ki-67 nuclear staining index [7]. Patients with recurrent hypoglycemia and newly diagnosed liver metastases were considered to have insulin-producing liver metastases. PHPT, PA, GEP-NETs, adrenal adenomas (AA), and thymic carcinoid tumors were diagnosed according to their corresponding guidelines [4, 8–11].

### Data collection

We retrospectively analyzed the detailed clinical information of the 55 patients and collected the following data: sex, age at hypoglycemia onset, age at diagnosis of MEN1 and insulinoma, family history, all MEN1-associated endocrine tumors, clinical signs, laboratory test results, imaging findings, pharmacological and surgical management regimens, post-treatment outcomes, type of *MEN1* gene mutation, pathological information, and results of oral glucose tolerance test (OGTT) performed with the presence of insulinoma in 51 patients requiring assessment of glucose metabolism.

Matsuda-insulin sensitivity index (ISI) was defined as 10,000/square root of [(mean plasma insulin × mean plasma glucose during OGTT) × (fasting plasma glucose × fasting plasma insulin)] [12].

### Laboratory assessment

The glucose oxidase assay was used to determine serum glucose levels. Serum insulin and C-peptide levels were examined using radioimmunoassay (DPC, USA) up to 1991 and chemiluminescence assays (ADVIA Centaur XP, Siemens) after 1991. Biochemical indices, including serum Ca, phosphorus (P), and albumin, were measured using a Beckman Automatic Biochemical analyzer (AU5800; Beckman Coulter). Serum ionized-Ca levels were measured using a blood gas analyzer radiometer (ABL800 FLEX; Denmark). Serum parathyroid hormone (PTH) levels were measured using chemiluminescence (ADVIA Centaur, Siemens, Germany). The following were the normal reference ranges for indices: fasting serum glucose, 3.9–6.1 mmol/L; fasting serum insulin, 5.2–17.2 μIU/mL; fasting serum C-peptide, 0.8–4.2 ng/mL; serum Ca, 2.13–2.70 mmol/L; serum iCa, 1.08–1.28 mmol/L; serum P, 0.81–1.45 mg/mL; serum albumin, 35–51 g/L; PTH, 13–65 pg/mL. Ca was corrected using the following formula: corrected CA = (0.8 × [normal albumin − patient’s albumin]) + serum Ca [13]. All tests were performed at the Clinical Laboratory of the PUMCH.

### Statistical analysis

Continuous data are expressed as mean ± standard deviation when normally distributed and as median (interquartile range) when non-normally distributed. Categorical variables are expressed as absolute numbers and percentages. The t-test was performed on the mean of normally distributed continuous data, whereas the Mann–Whitney U test was used for non-normally distributed continuous data. The chi-squared test was used to compare data between groups. Multiple linear regression analysis was used to explore the association between serum Ca and insulin/glucose ratio during hypoglycemic episodes. Adjustment for body mass index (BMI) or ISI, respectively, were conducted simultaneously. Statistical analyzes were performed using IBM SPSS Statistics for Windows version 26 (IBM Corp., Armonk, NY, USA). A two-tailed *P* < 0.05 was considered significant.

## Results

### Clinical and demographic characteristics of the study population

Among the 55 patients with MEN1-related insulinoma, the mean age at the first episode of HH was 30.76 ± 14.03 years (range 9–75 years), and that at the time of diagnosis of insulinoma and MEN1 was 34.49 ± 14.99 and 36.20 ± 14.08 years, respectively (Table 1). In 38 (69.1%) cases, the primary pancreatic lesions were multifocal pNETs, ranging from 0.9 to 10 cm in maximum diameter. Of the lesions, 38.2% were in the head/neck region only, 34.5% in the body/tail region only, and 27.3% in the head/neck and body/tail regions. One female patient had liver metastasis at the time of diagnosis. Among the 24 (43.6%) patients who underwent genetic testing, 23 (95.8%) were positive for MEN1 mutations, with mutations most common in exon 2 (21.7%, 5/23) and exon 3 (13%, 3/23) (Additional file 1: Table S1). Among the remaining 31 patients who did not receive genetic testing, 26 patients lacked blood samples because we had not yet performed routine genetic testing during their hospitalization (before 2010), and 5 declined because the diagnosis of MEN1 was already clear.

**Table 1:** Characteristics of MEN1 patients with insulinoma

	Overall (n = 55)
Gender, male, n (%)	29 (52.7%)
Height, cm, mean ± SD	165.04 ± 9.71
Weight, kg, mean ± SD	68.63 ± 13.49
BMI, kg/m^2^ mean ± SD	25.16 ± 4.15
Age, years, mean ± SD at	
Initial hypoglycemia	30.76 ± 14.03
Insulinoma diagnosis	34.49 ± 14.99
MEN1 diagnosis	36.20 ± 14.08
Delay of diagnosis of, years, median (IQR)	
Insulinoma	2 (1–5)
MEN1	4 (1–10)
Follow-up period, years, mean ± SD	5.75 ± 5.31
During hypoglycemic episodes, mean ± SD	
Glucose, mmol/L	2.25 ± 0.56
Insulin, μIU/mL	23.77 (10.52–47.82)
C-peptide, ng/mL	3.38 ± 1.79
INS/GLU	8.54(4.21–20.45)
C-peptide/GLU	1.53 ± 1.00
Ca, mmol/L, mean ± SD	2.61 ± 0.24
iCa, mmol/L, median (IQR)	1.33 (1.22–1.37)
Albumin, g/L, mean ± SD	42.19 ± 4.46
PTH, pg/mL, median (IQR)	110 (55.23–187.20)
P, mmol/L, median (IQR)	0.99 (0.86–1.29)
Matsuda-ISI, mean ± SD	73.72 ± 42.35
Number of pNETs, n (%)	
Multiple	38 (69.1%)
Single	17(30.9%)
Location of pNETs, n (%)	
Head/neck	21 (38.2%)
Body/tail	19 (34.5%)
Head/neck and Body/tail	15 (27.3%)
Maximum diameter of pNETs, cm, mean ± SD	2.41 ± 1.44
Lymph nodes removal at initial surgery, n (%)	8 (16%)
Lymph node metastases at initial surgery, n (%)	1 (2%)
Liver metastases	1 (2%)
Tumor grade, n (%)	
Grade 1	24 (63.2%)
Grade 2	14 (36.8%)
Histopathological Ki67% of surgery samples biopsy, %, mean ± SD	2.87 ± 1.94
Mitotic figures, %, median (IQR)	2 (1–3.25)

MEN-1, multiple endocrine neoplasia type 1; BMI, body mass index; INS, insulin; GLU, glucose; C-P, C-peptide; Ca, calcium; iCa, Ionized-Ca; PTH, parathyroid hormone; P, phosphorus; Matsuda-ISI, Matsuda-Insulin Sensitivity Index

### Hypoglycemic symptoms

All 55 subjects experienced recurrent hypoglycemic symptoms manifesting as neuroglycopenic symptoms (100%) and/or autonomic symptoms (81.8%). The most common neuroglycopenic symptoms included loss of consciousness or coma (85.5%) and seizures (10.9%). The most common autonomic symptoms were sweating (63.6%), palpitations (50%), hunger (34.2%), and dizziness (21.8%). hypoglycemia occurred most often during periods of fasting (98.2%), whereas it was rare after meals (9.1%). The mean minimum serum glucose level during hypoglycemic episodes was 2.25 ± 0.56 mmol/L, the median insulin level was 23.77 μIU/ml, and the mean C-peptide level was 3.38 ± 1.79 ng/mL (Table 1), and there was no association between these findings and tumor size.

### Distribution of endocrine lesions

A review of the course of disease course revealed that all patients were diagnosed with MEN1 after the discovery of insulinoma, and none of them were screened for MEN1 beforethe diagnosis of insulinoma, including three patients with a family history of MEN1. In addition, 13 (23.6%) patients with insulinoma only as their first presentation developed other manifestations of MEN1 after a median of 3 years (range 1–26 years). PHPT or PA was found concurrently with the onset of insulinoma in 15 (27.3%) and one (1.8%) patient, respectively, and the above three adenomas were discovered simultaneously in one (1.8%) patient. There was definite evidence of gastrinoma or PA only as of the first manifestation of MEN1 in one (1.8%) and three (5.5%) patient, respectively. The order of manifestations of adenomas was unclear in 21 (38.2%) patients (Additional file 1: Table S2).

**Fig. 1:**
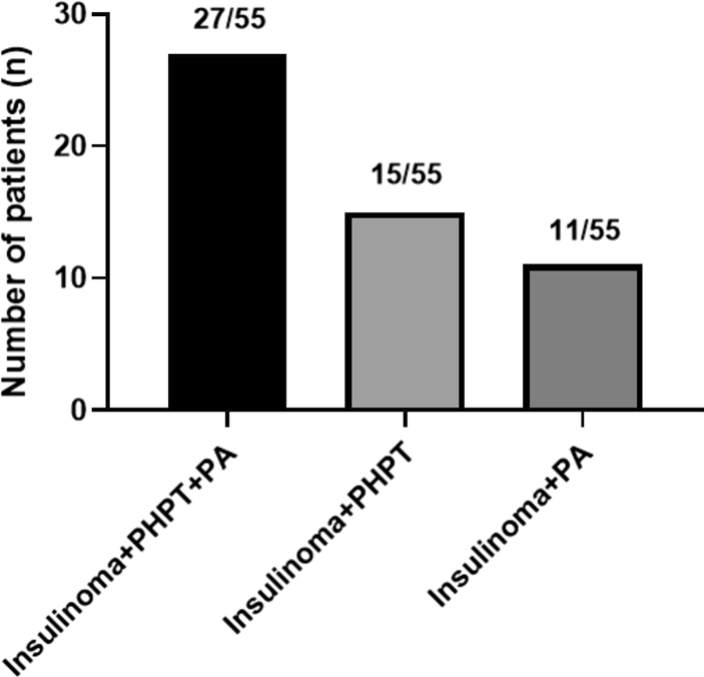
Distribution of endocrine lesions in our series of MEN1 patients. PHPT, primary hyperparathyroidism; PA, pituitary adenoma

As the disease progressed, all 55 patients developed adenomas of other glands, and the distribution of their clinical manifestations is depicted in detail in Fig. 1. In addition to insulinomas, PHPT (78.2%, 43/55) and PA (69.1%, 38/55) were the most common, followed by AA (16.4%, 9/55), gastrinomas (16.4%, 9/55), and gastroduodenal carcinoid tumors (1.8%, 1/55). The most frequent phenotypic combinations were insulinoma/PHPT/PA in 27 patients (49.09%), insulinoma/PHPT in 15 patients (27.3%), and insulinoma/PA in 11 patients (20%).

Among the patients with PA, 25 (65.8%) had non-functioning PA, 12 (31.6%) had prolactinomas, and one (2.63%) had both prolactin and growth hormone secretion. Seven (18.4%) patients with PA underwent surgery, and four (10.5%) received dopamine agonists for prolactinomas. Ten (18.2%) patients had adrenocortical tumors, and all adenomas in this group were non-functioning adenomas.

### Relationship between serum Ca and clinical characteristics

After excluding the 13 patients with missing serum Ca values when the insulinoma was untreated, compared with the 18 (42.9%) patients with normal serum Ca, no significant differences were found in serum glucose levels during hypoglycemic episodes in the 24 (57.1%) patients with hypercalcemia (Table 2). However, the latter group had significantly lower serum insulin concentrations (51.14 [33.65–71.54] vs 14.49 [6.83–23.15], *P* < 0.001) and insulin/glucose ratio (22.58 [13.68–29.98] vs 5.77 [2.71–13.1], *P* < 0.001) during hypoglycemic episodes. According to multiple linear regression analysis, serum Ca was negatively associated with insulin/glucose ratio after adjustment for BMI (B = − 26.621, 95% CI: − 45.944 to − 7.298, *P* = 0.008) or ISI (B = − 35.825, 95% CI: − 61.758 to − 9.891, *P* = 0.009). Patients with hypercalcemia had a tendency for older mean age at diagnosis and a larger diameter of the primary lesion, although no statistical difference was reached. After a median follow-up of 7 (range 1–19) years, 3 and 8 patients in the normocalcaemia and hypercalcemia groups, respectively, developed recurrent hypoglycemia. Four patients in the hypercalcemia group developed insulin-producing liver metastases, and the remaining seven had new insulinomas.

**Table 2:** Clinical characteristics of the patients according to serum Ca concentration

	Hypercalcemia n = 24	Normocalcemia n = 18	*P* value
Gender, male, n (%)	13 (59%)	9 (41%)	1
Height, cm, mean ± SD	164.19 ± 12.02	165.35 ± 8.66	0.738
Weight, kg, mean ± SD	66.38 ± 12.76	70.81 ± 14.1	0.265
BMI, kg/m^2^, median (IQR)	22.45 (20.49–26.88)	26.33 (23.67–28.6)	0.057
Ca, mmol/L, mean ± SD	2.75 ± 0.2	2.43 ± 0.13	< 0.001**
iCa, mmol/L, median (IQR)	1.35 (1.32–1.45)	1.21 (1.19–1.22)	0.002**
Albumin, g/L, mean ± SD	41.43 ± 4.93	43.94 ± 3.78	0.082
PTH, pg/mL, median (IQR)	147.35 (75.55–270)	48.3 (33.35–75.3)	0.001**
P, mmol/L, median (IQR)	0.94 (0.8–1.22)	1.04 (0.98–1.36)	0.044*
Age, years , mean ± SD at			
Initial symptoms	31.88 ± 16.36	28.17 ± 11.53	0.417
Insulinoma diagnosis	36.63 ± 17.06	31.67 ± 12.47	0.304
MEN1 diagnosis	38.50 ± 15.41	31.67 ± 12.47	0.131
Delay of diagnosis of, median (IQR), years			
Insulinoma	2 (1–6)	2.5 (1–3.25)	0.777
MEN1	6 (2.25–11)	2.5 (1–4.75)	0.045*
Hypoglycemia related indexes, mean ± SD			
Glucose, mmol/L	2.35 ± 0.59	2.18 ± 0.47	0.329
Insulin, μIU/mL	14.49 (6.83–23.15)	51.14 (33.65–71.54)	< 0.001**
C-peptide, ng/mL	3.09 ± 1.7	4.11 ± 1.78	0.085
INS/GLU	5.77 (2.71–13.1)	22.58 (13.68–29.98)	< 0.001**
C-P/GLU	1.38 ± 0.91	1.95 ± 1.04	0.081
Matsuda-ISI, mean ± SD	73.72 ± 42.35	59.68 ± 43.7	0.414
Number of pNETs, n (%)			
Multiple	20 (83.3%)	10 (55.6%)	0.104
Single	4 (16.7%)	8 (44.4%)	
Maximum diameter of pNETs, cm, mean ± SD	2.31 ± 1.17	1.92 ± 0.58	0.112
Tumor grade, n (%)			
Grade 1	8 (61.5%)	11(68.8%)	0.714
Grade 2	5 (38.5%)	5(31.2%)	
Histopathological Ki67% of surgery samples biopsy,%, mean ± SD	2.82 ± 1.83	2.22 ± 1.3	0.894
Mitotic figures,%, median (IQR)	1 (1–4)	2 (1–3)	0.776

BMI, Body Mass Index; Ca, calcium; iCa, Ionized-Ca; PTH, parathyroid hormone; P, phosphorus; MEN-1, multiple endocrine neoplasia type 1; INS, insulin; GLU, glucose; C-P, C-peptide; Matsuda-ISI, Matsuda-Insulin Sensitivity Index

^*^p < 0.05, **p < 0.01

**Fig. 2:**
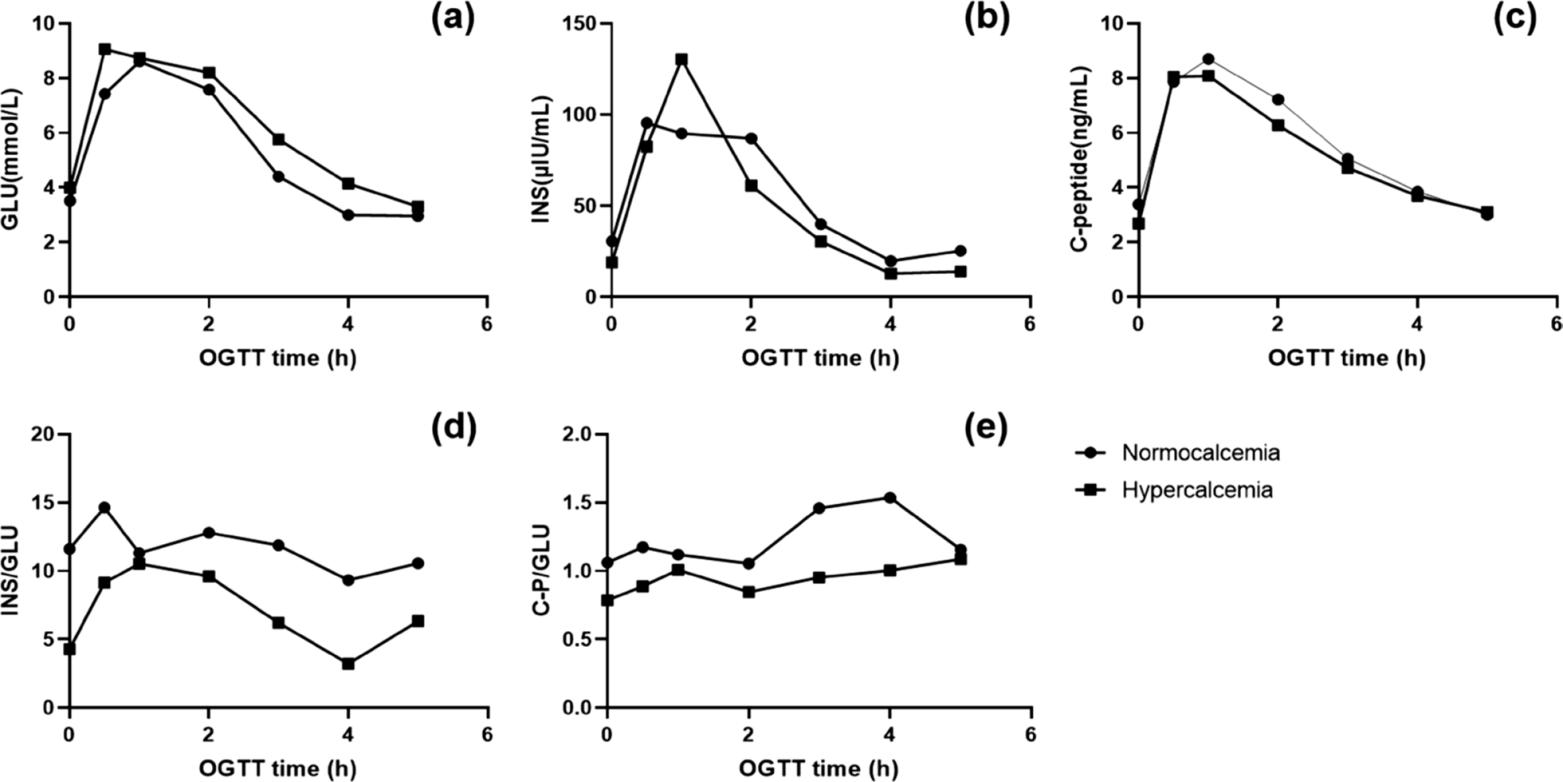
Results of OGTT in patients according to serum Ca concentration. OGTT, oral glucose tolerance test; INS, insulin; GLU, glucose; C-P, C-peptide. Serum glucose (**a**), insulin (**b**), C-peptide (**c**), INS/GLU ratio (**d**) and C-P/GLU ratio (**e**) values during the 5 h OGTT

In the oral glucose tolerance test (OGTT), the serum insulin, C-peptide, insulin/glucose ratio, and C-peptide/glucose ratio were lower at 0 h, 2 h, 3 h, 4 h, and 5 h in those with high serum Ca than in those with normal serum Ca, while serum C-peptide at 0.5 h and serum insulin at 1 h was higher in the former group, but the differences were not statistically significant (Fig. 2).

### Treatment and efficacy

Fifty (90.9%) patients underwent surgery (Table 3); three (5.5%) patients did not receive any treatment at our institution and were lost to follow-up. Insulinoma was localized in 16 patients (30.8%) who underwent typical resections; enucleation was performed in 10 patients and distal pancreatectomy was performed in six patients. In 36 patients (69.2%), the disease was considered multifocal and treated with combined pancreatectomy; most patients (55.6%, 20/36) underwent multiple enucleations, and nine had distal pancreatectomy with enucleation. Among the other two patients who did not undergo surgery, one patient received somatostatin analogs (SSAs) (four treatments with sandostatin LAR), and one underwent anhydrous alcohol injection to relieve hypoglycemic symptoms.

**Table 3:** Treatments of insulinomas in MEN1 patients

	Overall (n = 52)	Localized pNETs (n = 16)	Multifocal pNETs (n = 36)
Surgery, n (%)			
Enucleation	10 (19.2%)	10 (62.5%)	0
Multiple enucleations	20 (38.5%)	0	20 (55.6%)
Distal pancreatectomy	10 (19.2%)	6 (37.5%)	4 (11.1%)
Distal pancreatectomy and enucleation	9 (17.3%)	0	9 (25%)
Whipple/PPPD	1 (1.9%)	0	1 (2.8%)
Pharmacological treatment, n (%)			
Anhydrous alcohol injection	1 (1.9%)	0	1 (2.8%)
SSAs	1 (1.9%)	0	1 (2.8%)

PPPD, pylorus‐preserving pancreatoduodenectomy; SSAs, somatostatin analogs

All patients who underwent surgery were initially cured, and none developed persistent hypoglycemia after surgery. After a median postoperative follow‐up of 6 (range 1–19) years, 13 (26%) had recurrent hypoglycemia at a mean of 7.6 ± 4.27 years after surgery, nine (69.2%) of them had a new insulinoma and four (30.8%) developed insulin‐producing liver metastases, most of these patients (9/13, 69.2%) had had the multifocal disease and had undergone multiple enucleations. Eleven (84.6%) of these patients required a second operation, and two patients (15.4%) rejected the operation with extra meals every day. Of the 13 patients aged less than 22 years at the time of surgery, six developed recurrent insulinoma. In terms of complications, five patients who underwent distal pancreatectomy developed abnormal glucose tolerance or new-onset diabetes mellitus after an average of seven years.

### Pathology

Histopathological analysis was performed on samples following surgical resection in all patients. Immunohistochemical reports were not available for retrieval in three patients, and details of insulin staining and mitotic figures were absent in nine patients. Among the other 38 patients with complete pathological information, 13 (34.2%) patients had no insulin-positive pNET, three of whom had recurrent hypoglycemia 8.33 ± 7.02 years after initial surgical cure.

### *Clinical characteristics of patients aged* < *22 years*

In our cohort, 19 (34.5%) patients presented with initial symptoms of hypoglycemia before the age of 22 years, and the mean age at diagnosis of insulinoma and MEN1 was 19.89 ± 5 and 22.95 ± 5.79 years, respectively (Table 4). Seventeen (94.7%) patients underwent surgery that was immediately successful, one (5.3%) patient received anhydrous alcohol injections, and one (5.3%) patient was not treated. Eight patients (42.1%) had recurrent hypoglycemia at a mean of 8.14 ± 4.56 years after surgery, six (75%) of whom initially underwent multiple enucleations. The recurrence rate in patients aged > 22 years who underwent surgery was 13.9%, with no statistically significant difference between the two groups.

**Table 4:** Clinical characteristics of the subgroups by age

	< 22 years n = 19	> 22 years n = 36	*P* value
Gender, male, n (%)	11 (37.9%)	18 (63.1%)	0.777
Height, cm, mean ± SD	164.24 ± 10.06	165.53 ± 9.64	0.67
Weight, kg, mean ± SD	65.91 ± 10.4	70.29 ± 15	0.256
BMI, kg/m^2^, mean ± SD	24.42 ± 3.04	25.59 ± 4.66	0.359
Ca, mmol/L, mean ± SD	2.6 ± 0.17	2.61 ± 0.27	0.785
iCa, mmol/L, mean ± SD	1.29 ± 0.07	1.38 ± 0.17	0.199
Albumin, g/L, median (IQR)	43 (40.75–48.25)	41.5 (39.8–44)	0.063
PTH, pg/mL, median (IQR)	95.2 (49.6–145.85)	126.4 (56.8–262)	0.251
P, mmol/L, median (IQR)	1.02 (0.91–1.33)	0.99 (0.83–1.29)	0.625
Age, years , mean ± SD at			
Initial symptoms	16.89 ± 3.02	38.08 ± 11.82	< 0.001**
Insulinoma diagnosis	19.89 ± 5	40.72 ± 14.22	< 0.001**
MEN1 diagnosis	22.95 ± 5.79	42.91 ± 12	< 0.001**
Delay of diagnosis of, median (IQR), years			
Insulinoma	2 (1–4)	2 (1–5.75)	0.753
MEN1	5 (2–11)	3.5 (1–9.75)	0.689
Hypoglycemia related indexes, mean ± SD			
Glucose, mmol/L	2.24 ± 0.67	2.25 ± 0.49	0.942
Insulin, μIU/mL	31.2 (10.24–46.56)	22.53 (9.34–49.36)	0.776
C-peptide, ng/mL	3.66 ± 1.65	3.23 ± 1.88	0.463
INS/GLU	12.74 (4.21–17..88)	7.14 (3.68–25.2)	0.891
C-P/GLU	1.66 ± 1.1	1.45 ± 0.95	0.521
Matsuda-ISI, mean ± SD	47.46 ± 27.45	70.25 ± 44.51	0.081
Number of pNETs, n (%)			
Multiple	15 (78.9%)	23 (63.9%)	0.4
Single	4 (21.1%)	13 (36.1%)	
Maximum diameter of pNETs, cm, mean ± SD	2.23 ± 0.62	2.5 ± 1.73	0.883
Tumor grade, n (%)			
Grade 1	5 (55.6%)	19 (65.5%)	0.7
Grade 2	4 (44.4%)	10 (34.5%)	
Histopathological Ki67% of surgery samples biopsy,%, mean ± SD	2.86 ± 1.95	2.63 ± 1.95	0.676
Mitotic figures,%, median (IQR)	2 (1–5)	2 (1–3.5)	0.69
Surgery, n (%)			
Enucleation	2 (10.5%)	8 (22.2%)	
Multiple enucleations	9(47.4%)	11 (30.6%)	
Distal pancreatectomy	3(15.8%)	7 (19.4%)	
Distal pancreatectomy and enucleation	3 (15.8%)	6 (16.7%)	
Whipple/PPPD	0	1 (2.8%)	
Pharmacological treatment, n (%)			
Anhydrous alcohol injection	1 (5.3%)	0	
SSAs	0	1 (2.8%)	
Untreated	1 (5.3%)	2 (5.6%)	

BMI, Body Mass Index; Ca, calcium; iCa, ionized-Ca; PTH, parathyroid hormone; P, phosphorus; MEN-1, multiple endocrine neoplasia type 1; INS, insulin; GLU, glucose; C-P, C-peptide; Matsuda-ISI, Matsuda-Insulin Sensitivity Index

**p < 0.01

## Discussion

MEN1 is an autosomal dominant endocrine neoplasia syndrome characterized by a high risk of neoplasia in the endocrine organs. In this retrospective observational study, we described the clinical characteristics of patients with MEN1-associated insulinoma in the Chinese population for the first time and found that the rates of postoperative recurrence and long-term complications in patients were significantly different from those in other international centers, and might be related to the surgical method. In addition, elevated serum Ca levels in patients with PHPT may affect insulin secretion.

In our series, there was no significant difference in sex, distribution (52.7% were male), which is different from previous MEN1 studies, which had more female patients (64.2%) [14]. Furthermore, 69.1% (38/55) of our patients presented with multifocal pNETs, confirming the trend of the multifocality of pNETs in MEN1 [15, 16] and higher than the 30% reported in the previous studies [3]. In contrast to the previously described frequency of metastatic disease in MEN1, we observed a higher frequency of metastases with an incidence of 9.1% (5/55) [14]. The mean age at diagnosis of MEN1 and insulinoma in our patients was similar to that reported in studies [17, 18]. Affected patients mostly present with neuroglycopenic symptoms caused by higher insulin and proinsulin secretion [19]. Similar to previous reports, we did not find a correlation between the severity of hypoglycemic symptoms and tumor size [20].

In addition, the incidence of PHPT was only 78.2%, which is difficult to compare because of the lack of data related to MEN1 patients with insulinoma from other centers, and 32.7% of affected patients were symptomatic. Moreover, insulinoma was the first manifestation in 13 (23.6%) patients, and it was detected simultaneously with PHPT or PA in 17 (30.9%) patients. These results emphasize that PHPT is not always the first biological or clinical abnormality to emerge in the course of MEN1 disease [14, 21, 22] and may explain why the prevalence of PHPT in our cohort may yet be low. In contrast, PHPT was diagnosed at an older age (35.79 ± 14.19 years) than insulinoma, and the mean age of patients with asymptomatic PTHT (95.4%, 41/43) that was detected only by laboratory testing of serum PTH and Ca was even older (36.15 ± 14.32 years). Therefore, it is important to screen for MEN1 in patients with insulinoma, especially younger patients, and to obtain a correct diagnosis before the appearance of overt symptoms.

Previous studies have found that Ca levels in the circulation were elevated in patients with insulinoma following a selective arterial calcium injection test and may stimulate insulinoma secretion [23] and its mechanism may be related to the calcium-sensing receptor (CaSR) gene [24]. In addition, another study of patients with PHPT reported increased insulin secretion and decreased sensitivity to both basal and stimulated insulin [25]. In our study, there were differences in insulin secretion between patients with different serum Ca levels. First, in contrast to the results of the above mentioned papers, patients with high serum Ca levels had significantly lower insulin and insulin/glucose ratios during hypoglycemic episodes, which is not associated with insulin resistance and requires further comparative studies in other MEN1 populations. Second, patients with hypercalcemia exhibited a trend toward older mean age at diagnosis, a larger diameter of primary lesions, and a higher incidence of recurrent hypoglycemia. These results suggested that the prognosis in the high serum Ca group may have been relatively poor, although the differences described in the second point were not statistically significant.

However, during the OGTT, serum C-peptide level at 0.5 h and serum insulin level at 1 h was higher in those with high serum Ca than in those with normal serum Ca. This could be related to the mechanism by which serum Ca promotes insulin secretion in response to nutrient stimulation. Extracellular iCa influx through voltage-dependent iCa channels leads to an increase in cytoplasmic iCa concentration and initiates iCa-dependent cell division containing insulin-secreting granules [26]. Glucose-induced insulin secretion (GIIS) follows a biphasic time course consisting of an initial transient response lasting less than 10 min (phase I secretion) followed by a phase II (sustained) response that is below the peak insulin release of phase I but still tenfold higher than the basal secretion [27]. Therefore, we hypothesized that insulin secretion after feeding differs between hypercalcemic and normocalcaemic individuals, with increased insulin secretion in the first phase in the hypercalcemic group, possibly related to the stimulation of β-cell secretion because of high serum Ca levels and greater sensitivity of this response to nutrient stimulation.

In our cohort, 19 (34.5%) patients developed hypoglycemic symptoms before the age of 22 years. The mean size of resected tumors in our study was similar compared to other cohorts of young patients with MEN1-associated insulinomas [17, 28], but we had a higher rate of postoperative recurrence, which may be related to the selection of surgical options. In addition, the later mean age of diagnosis in our patients may also be related to prognosis, as it was claimed that earlier diagnosis improves the outcome [29, 30].

Surgical resection is the only curative treatment for insulinoma and is recommended regardless of tumor size. Current guidelines recommend an individualized treatment approach depending on the location and size of the suspected insulinoma [31]. For isolated tumors or one dominant tumor, enucleation or segmental pancreatic resection of the dominant tumor is usually performed at our center to minimize surgical trauma and preserve normal pancreatic tissue. The feasibility of resection depends on the size and location of the insulinoma and its relationship with the main pancreatic duct [18]. The majority of patients with multifocal disease underwent multiple enucleations (55.6%, 20/36), and 45% (9/20) had a postoperative recurrence, which was a higher rate than that reported in other centers where distal pancreatectomy and enucleation were mainly performed; however, we had a lower incidence of pancreatic insufficiency [18]. Patients with localized lesions mainly underwent enucleation, and the rate of postoperative recurrence was similar to that of patients who primarily underwent distal pancreatectomy at other centers. Thus, based on the current findings, a more aggressive treatment approach for patients with multiple lesions seems necessary, and the localization of insulinoma in these patients is particularly important. During the time of preoperative imaging, surgical decisions in patients with multifocal lesions should be adapted to the needs of the patient and guided by the location of the insulinoma [31]

Genetic testing was conducted in 24 of our patients after the onset of the disease, and it was possible to identify 23 carriers of *MEN1* gene mutations, which were observed in exons 2, 3, 4, 5, 6, 7, 8, 9, 10, and introns 3 and 4, with the most frequently observed mutation site being exon 2. The distribution of mutant loci in our patients was similar to that reported in a previous study in Japan [32], although the results differed from those reported by Turner [33]. Unfortunately, no significant genotype–phenotype or genotype-prognosis correlations were observed in this study, which is consistent with the results of other MEN1 series [14, 32, 34–36]. Furthermore, a study on a French cohort showed that the survival rate of patients with mutant MEN1 was significantly lower in carriers of MEN1 mutations affecting the binding site to JunD33, presenting a twofold higher risk of death from MEN1-associated tumors.

The present study has several limitations. First, the study involved a single institution, and the number of patients (55) was insufficient to draw firm conclusions from the results. Second, the follow-up period was relatively short. Third, the long enrolment period of nearly two decades (January 2002 to August 2021) implies a lack of uniformity in the diagnostic criteria, imaging resources, and treatment options. Fourth, only < 50% of patients underwent genetic testing, which limited further analysis of genotype–phenotype correlations. Fifth, the information on insulin staining of samples after surgical resection was limited, whereas it would be more reliable in confirming the removal of insulinoma using insulin staining.

In conclusion, we established an insulinoma cohort and differed from other similar studies in that our patients had a higher rate of postoperative recurrence, which may be related to the selection of the surgical method. In addition, the incidence of PHPT in our patients is probably lower, and similar analyzes in other study centers are needed for comparison. Moreover, individuals with high serum Ca may obscure the symptoms of hypoglycemia by producing less insulin during fasting than those with normal serum Ca; hence, they should be more alert to the occurrence of HH.

## Supplementary Information

**Additional file 1: Table S1.** The site and distribution of the MEN1 mutation; **Table S2.** First manifestation of MEN1 patients with insulinoma.

## Data Availability

The datasets used in the current study are not publicly available, but are available from the corresponding author on reasonable request.

## Abbreviations

AA: Adrenal adenomas
BMI: Body mass index
Ca: Calcium
CaSR: Calcium-sensing receptor
CT: Computed tomography
C-P: C-peptide
GEP: Gastro-entero-pancreatic
GEP-NETs: Neuroendocrine tumors of the functional gastro-entero-pancreatic tracts
GIIS: Glucose-induced insulin secretion
GLU: Glucose
GLP-1R: Glucagon-like peptide-1 receptor
HH: Hyperinsulinemic hypoglycemia
iCa: Ionized calcium
ISI: Insulin sensitivity index
INS: Insulin
Matsuda-ISI: Matsuda-insulin sensitivity index
MEN1: Multiple endocrine neoplasia type 1
NETs: Neuroendocrine tumors
NF-NETs: Non-functioning neuroendocrine tumours
OGTT: Oral glucose tolerance test
P: Phosphorus
PA: Pituitary adenoma
PHPT: Primary hyperparathyroidism
pNET: Pancreatic neuroendocrine tumor
PP: Pancreatic polypeptide
PPPD: Pylorus‐preserving pancreatoduodenectomy
PPT: Partial parathyroidectomy
PTH: Parathyroid hormone
SSAs: Somatostatin analogues
