# Supplementary material for: Analysis of 55 patients with multiple endocrine neoplasia type 1-associated insulinoma from a single center in China
Source: Orphanet J Rare Dis. 2022 Jun 13;17:219. doi: 10.1186/s13023-022-02370-1 (PMC9195405; doi:10.1186/s13023-022-02370-1)
Supplement: Supplementary file 1 — Additional file 1: Table S1. The site and distribution of the MEN1 mutation; Table S2. First manifestation of MEN1 patients with insulinoma. [file 13023_2022_2370_MOESM1_ESM.docx]

**Table S1. The site and distribution of the MEN1 mutation**

| Location | No. of patients | Phenotype |
| --- | --- | --- |
| Exon 2 |  |  |
| 249_252delGTCT | 1 | PHPT |
| 124 G＜A (p.G42S) | 1 | PHPT |
| G125T (p.G42V) | 1 | PHPT, PA |
| 122 T＜C | 1 | PHPT, PA |
| 313delC | 1 | PHPT, PA |
| Exon 3 |  |  |
| 593 G＜A (p.198WX) | 1 | PHPT, PA |
| 497 A＜C (p.Q166P) | 1 | PHPT, gastrinoma |
| 643-646 del ACAG | 1 | PHPT, gastrinoma, AA |
| Intron 3 |  |  |
| 783+1 G＜A | 1 | PHPT, gastrinoma, PA, AA |
| Intron 4 |  |  |
| 783+1 G＜A | 1 | PHPT, PA, AA |
| Exon 4 |  |  |
| 751delinsCC | 1 | PHPT, PA |
| Exon 5 |  |  |
| 839+2T＜G | 1 | PHPT, PA |
| Exon 6 |  |  |
| 839_840 insT | 1 | PHPT, PA, AA |
| 927+2T＜C | 1 | PHPT, PA |
| Exon 7 |  |  |
| 969 C＜A (Y323X) | 1 | PHPT, PA, AA, gastrinoma |
| Exon 8 |  |  |
| C1198T (p.Q400X) | 1 | PHPT, PA, gastrinoma |
| G1177T (p.E393X) | 1 | PHPT |
| Exon9 |  |  |
| 1213 delC (p.Q405Rfs) | 1 | PHPT, gastrinoma |
| Exon 10 |  |  |
| 1533delC (p.T511fs) | 1 | PHPT, PA, gastrinoma |
| 1561dupC (p.Arg521fs) | 1 | PHPT |

PHPT, primary hyperparathyroidism; PA, pituitary adenoma; AA, adrenal adenoma.

**Table S2. First manifestation of MEN1 patients with insulinoma**

| First manifestation | Overall (n=55) |
| --- | --- |
| Insulinoma, n (%) | 13 (23.6%) |
| Insulinoma+PHPT, n (%) | 15 (27.3%) |
| Insulinoma+PA, n (%) | 1 (1.8%) |
| Insulinoma+PHPT+PA, n (%) | 1 (1.8%) |
| Gastrinoma, n (%) | 1 (1.8%) |
| PA, n (%) | 3 (5.5%) |
| Uncertain, n (%) | 21 (38.2%) |

MEN-1, multiple endocrine neoplasia type 1; PHPT, primary hyperparathyroidism; PA, pituitary adenoma.
